# Five-year outcomes in patients with multivessel coronary artery disease undergoing surgery or percutaneous intervention

**DOI:** 10.1038/s41598-024-53905-4

**Published:** 2024-02-08

**Authors:** Szymon Jonik, Shigetaka Kageyama, Kai Ninomiya, Yoshinobu Onuma, Janusz Kochman, Marcin Grabowski, Patrick W. Serruys, Tomasz Mazurek

**Affiliations:** 1 1st Department of Cardiology, Medical University of Warsaw, Banacha 1a Str, 01-267 Warsaw, Poland; 2 Department of Cardiology, National University of Ireland, University Road Galway, Galway, H91 TK33 Ireland

**Keywords:** Interventional cardiology, Outcomes research

## Abstract

The outcomes from real-life clinical studies regarding the optimal revascularization strategy in patients with multivessel coronary artery disease (MVD) are still poorly investigated. In this retrospective study we assessed 5-year outcomes: primary, secondary endpoints and quality of life of 1035 individuals with severe coronary artery disease (CAD) treated either with coronary artery bypass grafting (CABG)—356 patients or percutaneous coronary intervention (PCI)—679 patients according to the recommendation of a local Heart Team (HT). At 5 years no significant difference in overall mortality and rates of myocardial infarctions (MI) were observed between CABG and PCI cohorts (11.0% vs. 13.4% for PCI, P = 0.27 and 9.6% vs. 12.8% for PCI, P = 0.12, respectively). The incidence of major adverse cardiac and cerebrovascular events (MACCE), mainly driven by increased rates of repeat revascularization (RR) were higher in PCI-cohort than in CABG-group (56.1% vs. 40.4%, P < 0.01 and 26.8% vs. 12.6%, P < 0.01, respectively), while CABG-patients experienced stroke more often (7.3% vs. 3.1% for PCI, P < 0.01). In real-life practice with long-term follow-up, none of the two revascularization modalities implemented following HT decisions showed overwhelming superiority: occurrence of death and MI were similar, rates of RR favoured CABG, while incidence of strokes advocated PCI.

## Introduction

Coronary artery disease (CAD) still remains one of the major problems of nowadays medicine with considerable impact of morbidity, mortality and healthcare systems. It is estimated that in 2020 CAD affected 244.1 million of people worldwide and was the leading cause of cardiovascular (CV) mortality accounted for 9.44 million of deaths and 185 million of disability-adjusted life years (DALYs) in 2021^1,2^. With an increasing number of treatment modalities, minimally-invasive methods of cardiac surgery, the enormous development of percutaneous strategies and the availability of new drugs improving angina symptoms and overall survival, CAD still significantly reduces life expectancy and worsens its quality. Therefore, an idea of multidisciplinary Heart Team (HT) for the management of the most burdened patients has been implemented and permanently plays a principal role in the real-life care of individuals with multivessel coronary artery disease (MVD)—class I recommendation in European and American guidelines^3–7^. Among numerous debates concerning CAD, one of the hottest was about whether coronary artery bypass grafting (CABG) or percutaneous coronary intervention (PCI) is the most suitable for patients with MVD. At each milestone in percutaneous techniques, PCI has been compared against the “gold standard” of surgery with respect to impact on mortality and quality of life. So far, many randomized-controlled clinical trials (RCTs) comparing outcomes of MVD-patients treated with CABG or PCI have been conducted, generally showing the superiority of CABG over PCI, however the debate is still far from the end^8–13^. The problem is more complicated, because in everyday clinical practice we have to deal with individual, severely burdened patients for whom relying solely on RCTs results is often insufficient. The outcomes of CABG or PCI are largely dependent on many different factors, the most important of which are: age, coronary lesion complexity, the extension of peripheral atherosclerosis, the severity of heart failure (HF), chronic kidney disease (CKD), diabetes or the presence of three-vessel disease (3-VD) or significant left main disease (LMD). Therefore, a clear indication which strategy is better for individual patient is not possible without considering the whole clinical presentation, availability of both methods and the local experience. Another pressing issue is the fact that many patients we have to deal with in everyday clinical practice (i.e. elderly, frailty or with active cancer) are often excluded from ongoing trials due to rigorous inclusion and exclusion criteria. Thus, to properly select the optimal mode of revascularization for MVD-patients, the need for studies based on real-life conditions seems more urgent. This kind of approach represents by Heart Team (HT) plays a pivotal role in the comprehensive management of CAD, with a particular focus on the most complex cases. In our study, we presented long-term outcomes and quality of life of patients with severe CAD (3-VD or/and LMD) hospitalized in tertiary cardiovascular care center and qualified after careful HT discussion to revascularization either with CABG or PCI and subsequent optimal medical therapy (OMT). By presenting this work, we would like to emphasize the importance of the outcomes derived from daily clinical practice and honour the concept of HT as fundamental link between RCTs and real-world evidence studies.

## Patients and methods

**Figure 1:**
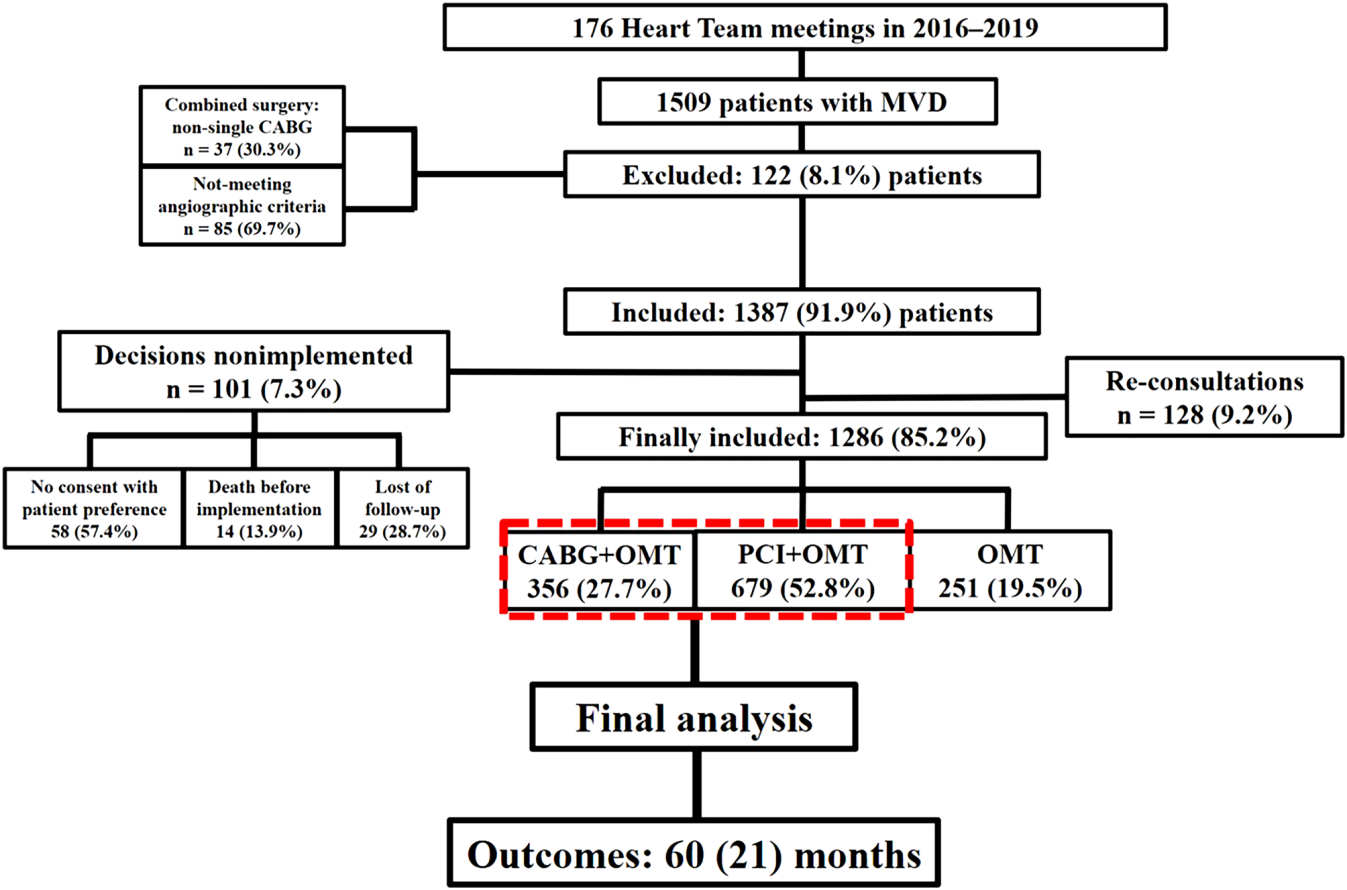
The main outline of the study. *MVD* multivessel coronary artery disease, *CABG* coronary artery bypass grafting, *PCI* percutaneous coronary intervention, *OMT* optimal medical therapy.

The detailed methods and study plan have already been described earlier^14^. Briefly, this single-center observational study was conducted in the 1st Department of Cardiology, Medical University of Warsaw, a large tertiary cardiovascular care center in Poland. The inclusion criteria were: aged ≥ 18 years and complete clinical, echocardiographic and angiographic characteristics. The exclusion criteria included the following: pregnancy/lactation, disseminated neoplastic process, life expectancy < 1 year and lack of informed, written consent^14^. All of patients were evaluated by a HT composed of interventional cardiologists, cardiac surgeons, clinical cardiologists and non-invasive imaging specialists and qualified to one of three main strategies: CABG with OMT, PCI with OMT or OMT alone^14^. Out of a total number of 1509 patients consulted for severe CAD during 176 HT meetings in 2016–2019, we performed final analysis for 1035 individuals meeting angiographic criteria of severe CAD (3-VD or/and LMD) and qualified after HT discussion to revascularization strategy: either with CABG (356 patients) or PCI (679 patients). Outcomes of OMT-cohort was described previously^14^. After implementing surgery of percutaneous approach all of patients were followed with OMT defined as using of drugs with proven impact on survival or reducing symptoms of CAD—aspirin or dual antiplatelet therapy (DAPT), angiotensin-converting enzyme inhibitors (ACEi) or angiotensin receptor blockers (ARBs), beta-blockers (BB), statins and aldosterone antagonists. The definitions of chronic kidney disease (CKD), severe pulmonary arterial hypertension (PAH) and anemia were also described previously^14^. 3-VD was defined as stenosis greater than 70% or between 40 and 70%, but assessed with functional tests (fractional flow reserve (FFR), instantaneous wave-free ratio (iwFR) or quantitative flow ratio (QFR)) as haemodynamically significant in at least three vessels with 1.5 mm or more in diameter, while the LMD was defined as LM stenosis equal or more than 50%. An overall mortality was primary endpoint, while major adverse cardiac or cerebrovascular events (MACCE) [i.e. overall mortality, stroke, myocardial infarction (MI), or repeat revascularization (RR)] and separate components of MACCE were defined as secondary endpoints. MI was defined using Fourth Universal Definition of Myocardial Infarction, stroke as clinical signs of local or global disturbance of cerebral function, lasting more than 24 h or leading to death, with no evident cause other than of vascular origin, while RR as repeat PCI or bypass graft placement for restenosis at the lesion treated during baseline revascularization. The mean (SD) follow-up was equal to 60 (21) months and ended on 31th of December, 2022. The main outline of the study was presented in Fig. 1. Additionally, general health status, using the short-form-36 (SF-36) questionnaire (totally and separately for physical component summary [PCS] and mental component summary [MCS]) before CABG or PCI and at the end of follow-up (EOF) for all alive participants was assessed. According to the Polish version of the questionnaire, with a maximum of 103 points for PCS and 68 points for MCS (171 points in total), the highest point value means the lowest quality of life, while the lowest point value indicates the highest level of quality of life. All experimental protocols, if undertaken, were approved by Medical University of Warsaw. An informed consent was obtained from all participating subjects or their legal guardians. All experiments and analyses were performed in accordance with the relevant guidelines and regulations. The normality of distribution for continuous variables was confirmed with the Shapiro–Wilk test. Categorical data were expressed as counts and percentages, while continuous data were presented as means with standard deviation (SD). Comparisons between groups were performed using the Pearson’s Chi-squared test for categorical variables, and Student’s *t* test or Mann–Whitney *U* test for unpaired continuous variables, and Wilcoxon rank-sum test for paired variables, according to data distribution. To compare the outcomes for all strategies with each other the hazard ratios (HRs) with 95% confidence intervals (95% CI) were calculated. Time to event analysis was performed using Kaplan–Meier curves. All *P* values were given to at least 2-sided and *P* value lower than 0.01 were considered statistically significant. The PQStat software (version 1.6.6, PQStat, Poznań, Poland) was used for statistical analysis.

## Results

### Study population

From January 2016 to December 2019, 176 HT meetings were held and a total of 1035 patients with severe CAD (3-VD or/and LM disease) meeting inclusion and exclusion criteria qualified by HT to CABG or PCI with subsequent OMT were followed up for a mean (SD) of 60 (21) months. The mean (SD) age of overall cohort was 68.2 (9.9) years, 75.7% were men and 9.5% presented with frailty syndrome. Regarding periprocedural risk, the mean (SD) values of EuroSCORE II [European System for Cardiac Operative Risk Evaluation II] and STS [Society of Thoracic Surgeons] score were 5.3 ± 3.2% and 3.4 ± 1.9%, respectively. Approximately 30% of patients had medically treated diabetes, of whom about one third required insulin. The prevalence of hypertension, dyslipidemia and current smoking was relatively high in overall cohort—83.9%, 81.2% and 19.0%, respectively. There were no significant differences between ratios of hypertension and dyslipidemia between the two groups. 10.7% of participants had hemodynamically significant stenosis of carotid arteries, while 8.3% had experienced a previous stroke. As regards statistically significant differences in co-morbidities between CABG and PCI groups, patients qualified for PCI were generally more burdened and presented more often with severe PH (10.8% vs. 4.8%, P < 0.01), CKD (30% vs. 18.5%, P < 0.01), atrial fibrillation (AF) [27.8% vs. 18.8%, P < 0.01], anemia (35.8% vs. 25.0%, P < 0.01), peripheral artery disease (PAD) [7.2% vs. 3.9%, P = 0.04)] and active cancer (4.0% vs 0.0%, P < 0.01). Furthermore, 42.2% of participants presented with acute coronary syndromes (ACS), of whom 3.2% with cardiogenic shock, while rest of them had chronic symptoms of CAD with more severe angina symptoms (Canadian Cardiovascular Society (CCS) class III-IV) in CABG-cohort. The rates of ST-segment elevation myocardial infarction (STEMI) and cardiogenic shock were higher in PCI-group (17.4% vs. 0.8%, P < 0.01 and 4.3% vs. 1.1%, P < 0.01, respectively), while the rate of non-STEMI (NSTEMI) in CABG-patients (41.9% vs. 24.6%, P < 0.01). The history of previous myocardial infarction (MI) was similar between both groups, while rate of prior revascularization was lower in CABG-patients (31.2% vs 42.4%, P < 0.01), both with history of previous PCI (25.6% vs. 32%, P = 0.03) and CABG (5.6% vs. 10.5%, P < 0.01). The prevalence of HF was more common in PCI-cohort (73.3% vs. 66.3%, P = 0.02), further these patients had also lower left ventricle ejection fraction (LVEF) [36.9% vs. 39.0%, P < 0.01]. The severity HF symptoms’ (New York Heart Association (NYHA) class III-IV) was similar between both groups. Complete revascularization was achieved in 60.9% of patients, more frequently in CABG-group (65.4% vs. 58.5% for PCI; P = 0.03). Baseline clinical characteristics (overall and by groups) in details was presented in Table 1.

**Table 1:** Baseline clinical characteristics.

Characteristics	Overall (1035)	CABG (356)	PCI (679)	P value
Preprocedural				
Age, years; mean (SD)	68.2 (9.9)	66.9 (9.2)	68.9 (10.1)	< 0.01
Gender, male; n (%)	784 (75.7)	289 (81.2)	495 (72.9)	< 0.01
BMI, kg/m^2^; mean (SD)	28.1 (3.6)	28.0 (3.3)	28.1 (3.7)	0.41
Fraility; n (%)	98 (9.5)	9 (2.5)	89 (13.1)	< 0.01
Current smoking; n (%)	197 (19.0)	65 (18.3)	132 (19.4)	0.65
COPD; n (%)	99 (9.6)	29 (8.1)	70 (10.3)	0.26
Diabetes; n (%)	317 (30.6)	98 (27.5)	219 (32.3)	0.12
With insulin; n (%)	105 (10.1)	29 (8.1)	76 (11.2)	0.12
Hypertension; n (%)	868 (83.9)	291 (81.7)	577 (85.0)	0.18
Severe PH; n (%)	90 (8.7)	17 (4.8)	73 (10.8)	< 0.01
Dyslipidemia; n (%)	840 (81.2)	284 (79.8)	556 (81.9)	0.41
Heart failure; n (%)	734 (70.9)	236 (66.3)	498 (73.3)	0.02
LVEF; % (SD)	37.7 (10.6)	39.0 (10.6)	36.9 (10.6)	< 0.01
NYHA class III-IV; n (%)	312 (30.1)	95 (26.7)	217 (32.0)	0.08
CKD; n (%)	270 (26.1)	66 (18.5)	204 (30.0)	< 0.01
Atrial fibrillation; n (%)	256 (24.7)	67 (18.8)	189 (27.8)	< 0.01
Anemia; n (%)	332 (32.1)	89 (25.0)	243 (35.8)	< 0.01
Prior MI; n (%)	507 (49.0)	189 (53.1)	318 (47.0)	0.06
Prior revascularization; n (%)	399 (38.6)	111 (31.2)	288 (42.4)	< 0.01
Prior PCI; n (%)	308 (29.8)	91 (25.6)	217 (32.0)	0.03
Prior CABG; n (%)	91 (8.8)	20 (5.6)	71 (10.5)	< 0.01
CCS class III-IV; n (%)	431 (41.6)	165 (46.3)	266 (39.2)	0.03
ACS; n (%)	437 (42.2)	152 (42.7)	285 (42.0)	0.82
STEMI; n (%)	121 (11.7)	3 (0.8)	118 (17.4)	< 0.01
Non-STEMI; n (%)	316 (30.5)	149 (41.9)	167 (24.6)	< 0.01
Cardiogenic shock on admission; n (%)	33 (3.2)	4 (1.1)	29 (4.3)	0.01
PAD; n (%)	63 (6.1)	14 (3.9)	49 (7.2)	0.04
Carotid stenosis; n (%)	111 (10.7)	34 (9.6)	77 (11.3)	0.38
Prior stroke/TIA; n (%)	86 (8.3)	26 (7.3)	60 (8.8)	0.40
Ischemic stroke/TIA; n (%)	72 (7.0)	21 (6.0)	51 (7.5)	0.33
Hemorrage stroke; n (%)	14 (1.4)	5 (1.4)	9 (1.3)	0.92
Active cancer; n (%)	27 (2.6)	0 (0.0)	27 (4.0)	< 0.01
EuroSCORE II, %; mean (SD)	5.3 (3.2)	3.9 (1.2)	6.0 (3.7)	< 0.01
STS score, %; mean (SD)	3.4 (1.9)	2.5 (0.8)	3.9 (2.2)	< 0.01
Procedural				
Complete revascularization; n (%)	630 (60.9)	233 (65.4)	397 (58.5)	0.03

*CABG* coronary artery bypass grafting, *PCI* percutaneous coronary intervention, *BMI* body mass index, *ACS* acute coronary syndrome, *LVEF* left ventricle ejection fraction, *CCS* Canadian Cardiovascular Society, *NYHA* New York Heart Association, *TIA* transient ischemic attack, *MI* myocardial infarction, *PAD* peripheral artery disease, *CKD* chronic kidney disease, *COPD* chronic obstructive pulmonary disease, *PH* pulmonary hypertension, *EuroSCORE II* European System for Cardiac Operative Risk Evaluation II, *STEMI* ST-segment elevation myocardial infarction, *STS* Society of Thoracic Surgeons score.

### Angiographic parameters

Overall, the mean (SD) number of affected lesions was 4.3 (1.5), quarter of patients had significant LMD, 72.7% coronary stenosis involving bifurcation, 28.2% severe calcification and 16.5% at least one artery considered chronically occluded (chronic total occlusion, CTO). The mean SYNTAX score for overall cohort was 30.2 (6.4) points, significantly increased in CABG-patients (31.3 vs. 29.6 points for PCI, P < 0.01). LM disease was found more frequently in CABG-cohort (30.6% vs. 23.3% for PCI, P = 0.01), while PCI-patients had more calcified arteries (30.5% vs. 23.9% for CABG, P = 0.02)—Table 2.

**Table 2:** Angiographic parameters.

Angiographic parameters	Overall (1035)	CABG (356)	PCI (679)	P value
Number of lesion; mean (SD)	4.3 (1.5)	4.2 (1.4)	4.3 (1.5)	0.42
LM disease; n (%)	267 (25.8)	109 (30.6)	158 (23.3)	0.01
Bifurcation; n (%)	752 (72.7)	261 (73.3)	491 (72.3)	0.73
Severe calcification; n (%)	292 (28.2)	85 (23.9)	207 (30.5)	0.02
Total occlusion; n (%)	171 (16.5)	77 (21.6)	164 (24.2)	0.36
SYNTAX score; mean (SD)	30.2 (6.4)	31.3 (5.9)	29.6 (6.5)	< 0.01

*LM* left main, *SYNTAX* Synergy between PCI with Taxus and Cardiac Surgery, *CABG* coronary artery bypass grafting, *PCI* percutaneous coronary intervention.

### Medications on admission and at discharge

On admission usage of statins and ACEi was higher among PCI-patients as compared with CABG-cohort (91.9% vs. 80.6% and 68.5% vs. 55.9%, respectively, P < 0.01), whilst application ratios of ARBs and BB were similar between these two groups. At discharge OMT was administrated for all participants with a higher percentage taking aspirin, P2Y12 inhibitors, NOAC (Novel Oral AntiCoagulants), statins, ACE-inhibitors and aldosterone antagonists in PCI-group (P < 0.01), while VKA (Vitamin K Antagonists) was frequently prescribed for CABG-patients (P < 0.01). The usage of ARB, BB and loop diuretics was similar in both groups—detailed in Table 3.

**Table 3:** Medications on admission and at discharge.

Medications on admission	Overall (1035)	CABG (356)	PCI (679)	P value
Statin; n (%)	911 (88.0)	287 (80.6)	624 (91.9)	< 0.01
ACE inhibitor; n (%)	664 (64.2)	199 (55.9)	465 (68.5)	< 0.01
ARB; n (%)	209 (20.2)	76 (21.3)	133 (19.6)	0.50
Beta-blocker; n (%)	785 (75.8)	264 (74.2)	521 (76.7)	0.36

Medications at discharge	Overall (993)	CABG (339)	PCI (654)	P value
Aspirin; n (%)	933 (94.0)	302 (89.1)	631 (96.5)	< 0.01
P2Y12 inhibitors; n (%)	706 (71.1)	71 (20.9)	635 (97.1)	< 0.01
VKA; n (%)	52 (5.2)	29 (8.6)	23 (3.5)	< 0.01
NOAC; n (%)	238 (24.0)	52 (15.3)	186 (28.4)	< 0.01
Statin; n (%)	922 (92.9)	294 (86.7)	628 (96.0)	< 0.01
ACE inhibitor; n (%)	685 (69.0)	206 (60.8)	479 (73.2)	< 0.01
ARB; n (%)	229 (23.1)	85 (25.1)	144 (22.0)	0.28
Beta-blocker; n (%)	798 (80.4)	270 (79.6)	528 (80.7)	0.68
Loop diuretic; n (%)	662 (66.7)	228 (67.3)	434 (66.4)	0.78
Aldosterone antagonist; n (%)	230 (23.2)	43 (12.7)	187 (28.6)	< 0.01

*CABG* coronary artery bypass grafting, *PCI* percutaneous coronary intervention, *P2Y12 inhibitors* clopidogrel, prasugrel, ticagrelor, *ACE* angiotensin-converting enzyme, *ARB* angiotensin receptor blocker, *NOAC* novel oral anticoagulants, *VKA* Vitamin K antagonists.

### Outcomes

**Figure 2:**
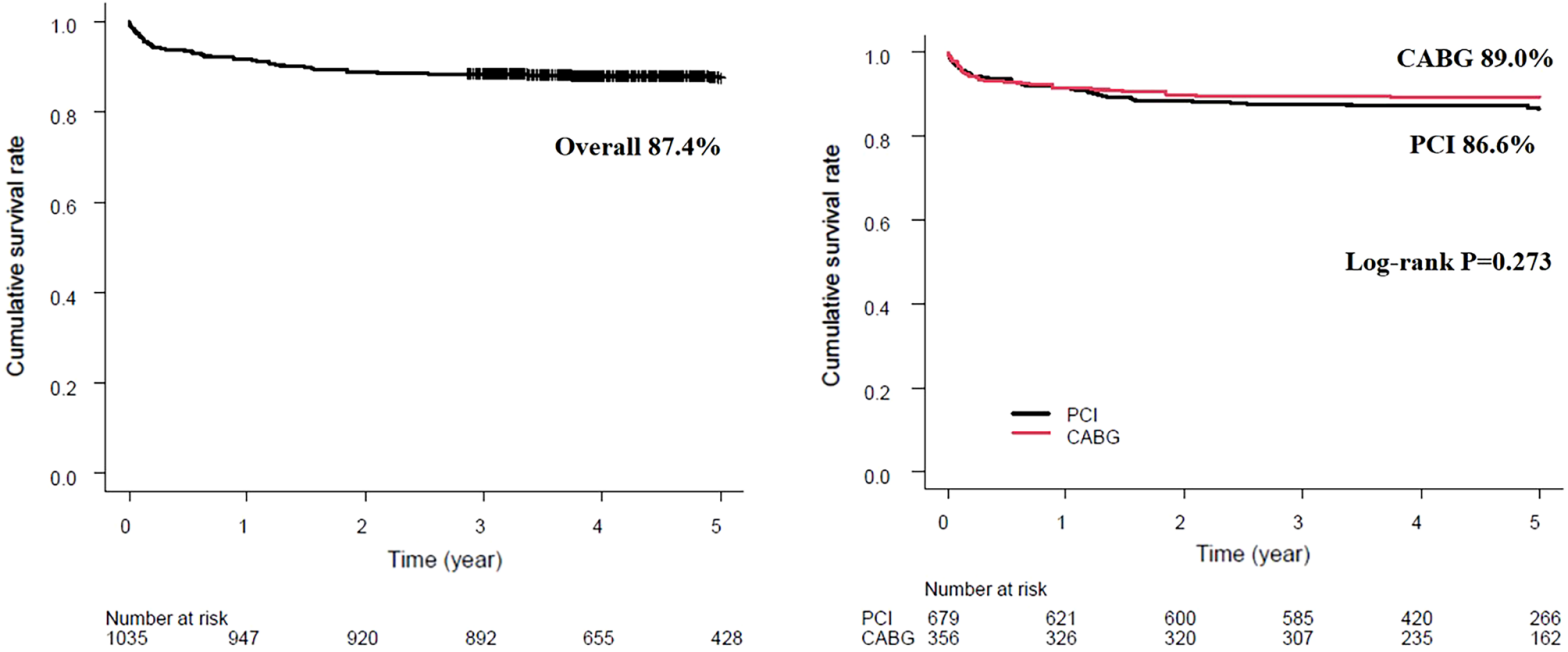
Primary endpoint—5-year mortality overall and in CABG– and PCI– cohorts. *CABG* coronary artery bypass grafting, *PCI* percutaneous coronary intervention.

In-hospital mortality was similar between CABG and PCI (17/356 (4.8%) vs. 25/679 (3.7%), respectively, P = 0.40). The all-cause death and MACCE in the overall cohort after 5 years was 130/1035 (12.6%) and 525/1035 (50.7%), respectively. The occurrence of primary endpoint did not significantly differ between CABG- and PCI-groups (39/356 (11.0%) vs. 91/679 (13.4%) for PCI, P = 0.27)—presented in Fig. 2. Compared to CABG, PCI was associated with increased rates of MACCE, mainly driven by higher rates of RR (381/679 (56.1%) vs. 144/356 (40.4%) for CABG, P < 0.01 and 182/679 (26.8%) vs. 45/356 (12.6%) for CABG, P < 0.01, respectively). The incidence of MI were similar between both strategies (34/356 (9.6%) vs. 87/679 (12.8%) for PCI, P = 0.12), while the inferiority of CABG for stroke was observed (26/356 (7.3%) vs. 21/679 (3.1%) for PCI, P < 0.01). Postprocedural hospital stay was significantly prolonged for CABG-patients (mean (SD): (9.9 (1.4) vs 4.3 (0.7) days for PCI, respectively; P < 0.01). The endpoints comparing CABG and PCI were detailed in Table 4.

**Table 4:** Primary and secondary endpoints.

Endpoints	Overall (1035)	CABG (356)	PCI (679)	P value
All-cause death, n (%)	130 (12.6)	39 (11.0)	91 (13.4)	0.27
MACCE, n (%)	525 (50.7)	144 (40.4)	381 (56.1)	< 0.01
MI, n (%)	121 (11.7)	34 (9.6)	87 (12.8)	0.12
Stroke, n (%)	47 (4.5)	26 (7.3)	21 (3.1)	< 0.01
Repeat revascularization, n (%)	227 (21.9)	45 (12.6)	182 (26.8)	< 0.01
In-hospital mortality, n (%)	42 (4.1)	17 (4.8)	25 (3.7)	0.40
Postprocedural hospital stay, days; mean (SD)	6.3 (1.0)	9.9 (1.4)	4.3 (0.7)	< 0.01

*MACCE* major adverse cardiac and cerebrovascular event, *CABG* coronary artery bypass grafting, *PCI* percutaneous coronary intervention, *MI* myocardial infarction.

### Quality of life

General health status assessed before implementing HT decisions—PCS, MCS and total—did not statistically differ between treatment strategies, while at the EOF patients treated with CABG achieved better improvement in PCS and total (P < 0.01), without significant differences in MCS values (P = 0.12)—Table 5.

**Table 5:** The quality of life before HT decisions and at the end of follow-up.

Before HT decisions	Overall (1035)	CABG (356)	PCI (679)	P value
PCS; mean (SD)	72.5 (18.3)	71.1 (18.2)	73.2 (18.4)	0.08
MCS; mean (SD)	51.9 (9.3)	51.6 (9.2)	52.0 (9.4)	0.49
Total; mean (SD)	124.3 (20.5)	122.7 (20.2)	125.2 (20.6)	0.06

End of follow-up	Overall (905)	CABG (317)	PCI (588)	P value
PCS; mean (SD)	65.9 (14.2)	63.9 (16.6)	67.0 (13.9)	< 0.01
MCS; mean (SD)	44.9 (9.8)	43.7 (9.6)	45.5 (9.9)	0.12
Total; mean (SD)	110.8 (16.0)	107.6 (18.3)	112.5 (14.9)	< 0.01

*CABG* coronary artery bypass grafting, *PCI* percutaneous coronary intervention, *PCS* physical component summary, *MCS* mental component summary.

### Logistic regression analysis

Moreover, to determine the factors independently related to increased incidence of primary endpoint we have performed multivariable, multinominal logistic regression analysis in the overall cohort of MVD-patients and additionally we performed an analysis of treatment effect for the primary outcome in prespecified subgroups. Our analysis revealed that (irrespectively of final treatment strategies—CABG + OMT or PCI + OMT): age, frailty, diabetes requiring insulin, COPD, severe PH, NYHA class III-IV, CKD, anemia, cardiogenic shock on admission, PAD, active cancer, EuroSCORE II, LM disease, number of lesion and SYNTAX score were independently associated with increased occurrence of overall mortality in long-term follow-up P < 0.01 for all). Regarding the most important parameters from baseline clinical and angiographic characteristics for 5-year of follow-up: for age < 65 years, female, diabetes and diabetes requiring insulin, reduced EF, non-STEMI, LM disease and increased SYNTAX score surgery was associated with improved prognosis (P < 0.01 for Odds Ratios), while PCI was better for frailty patients, with COPD and severe PH, after previous revascularization, MI and stroke/TIA, with STEMI, PAD, carotid stenosis, bifurcation and severe calcification (P < 0.01 for Odds Ratio).

## Discussion

Our retrospective study was designed to examine outcomes of patients with MVD qualified after HT evaluation to revascularization either with CABG or PCI with subsequent long-term OMT. When designing it, we asked to answer whether the results of our real-life study are consistent with the outcomes from large RCTs and could contribute important guidelines to current recommendations. Although the great advantage of HT decisions-based qualification process in large tertiary cardiovascular care center is nonrandomized conditions, this approach is not without its drawbacks. Given the fact that individuals who underwent PCI were older, more frailty and generally much burdened, similar mortality in both groups can be considered as hypothesis-generating. Although the patients were carefully discussed by an experienced HT, it should be deliberated whether the qualification process was always correct and whether the center's experience in both treatment strategies was similar. Also, the issue of hierarchy in the HT cannot be omitted, which has already been raised in the literature^15^. Given the advanced angiographic severity of CAD in PCI cohort, the rate of RR over a 5-year period equals 26.8% does not seem to be high. It needs to be highlighted that patients treated percutaneously had the increased rates of known predictors of restenosis: 30.6% had diabetes, mean lesions per patients was over four and lesions involving bifurcations, severely calcified or totally occluded were found in 72.3%, 30.5% and 24.2% individuals, respectively. Although, significantly lower incidence of RR was demonstrated in CABG-cohort, it did not translate into significant superiority in overall mortality or rates of MI. It is worth noting that the results of our real-life study in terms of increased RR rates in MVD-patients undergoing revascularization either with PCI or CABG are consistent with several meta-analyses conducted so far^16–18^. Furthermore, in two of these meta-analyses, increased RR rates in the PCI group did not translate into increased rates of MI or all-cause mortality^16,18^. However, in one study, the largest meta-analysis to date, outcomes from SYNTAX trial and other similar RCTs studies involving 11,518 patients comparing PCI with CABG for complex CAD revealed significantly higher rate of all-cause mortality in patients treated with PCI compared to CABG^19^. Subgroups analyses showed that in non-diabetic individuals with MVD and low (≤ 22) SYNTAX score, PCI was comparable to CABG in effectiveness and safety. Similarly, patients with non-complex LMD had similar survival either with PCI or CABG. However, in patients burdened with diabetes, a trend for better outcome with CABG was observed as the SYNTAX score increased. Results from this meta-analysis were adopted to current guidelines^20^. The changes in recommendations for myocardial revascularization led to a never-ending debate whether CABG or PCI was better for MVD-individuals^21–23^. The main reason for disputation was the lack of long-term follow-up in comparative PCI and CABG trials. Publication of the SYNTAXES (Synergy Between Percutaneous Coronary Intervention With TAXUS and Cardiac Surgery Extended Survival) study attempted to fill this gap, providing long-term outcomes of all-cause death for MVD-patients treated either with CABG or PCI with a median follow-up of 11.2 years^24^. The primary outcome of these trial was overall mortality at 10 years in individuals previously assigned to PCI or CABG. The secondary outcome was all-cause death at maximum available follow-up. There was no significant difference in primary endpoint between these two groups (27% vs 24% for PCI and CABG, respectively) with vital status available for 93% and 95% patients in PCI- and CABG-cohort, respectively (HR 1.17 [95% CI 0.97–1.41], P = 0.092)^24^. Further investigations showed that patients with 3-VD treated with CABG had better survival compared with PCI at 10 years (21% vs. 28% of all-cause death, respectively; HR 1.41; 95% CI 1.10–1.80; P = 0.006). In 3-VD, high SYNTAX score (> 33) was the principal differentiator. Contrary to previous reports in SYNTAX or similar studies in diabetic patients, diabetes status was not a differentiator of prognosis^25^. Furthermore, no difference between these two strategies was observed in LMD-patients (26% vs. 28% for CABG; HR 0.90; 95% CI, 0.68–1.20; P = 0.47). SYNTAXES is so far the longest study comparing outcomes of patients treated either with PCI or CABG in which 94% of patients had a complete long term follow-up. Unluckily, the primary endpoint of MACCE used in previous SYNTAX trial reports was not available due to the design of this study. Therefore, any deliberation on the exact cause of death reported in the SYNTAXES study is not possible^26^. Due to the end of the original SYNTAX trial, any reports after the 5 years of follow-up on medical treatment, invasive approach, or possible cross-over are lacking. Furthermore, outcomes from this more than 10-year-old study are inconsistent with current practice, including use of first-generation DES that are known to have higher RR and stent thrombosis rates^27^, incomplete revascularization in many patients, no intracoronary imaging, and a lack of physiological guidance for applied strategy. Majority of these limitations were dispelled in the SYNTAX II trial^28^. SYNTAX II strategy of incorporating both clinical and anatomical variables into HT decisions to guide myocardial revascularization led to better 5-year clinical outcomes in comparison with the SYNTAX trial, which evaluated anatomic factors only. Revascularisation in SYNTAX II was based on functional assessment, a third-generation stents was used, and the result was analysed and optimized using intravascular ultrasound (IVUS). For this trial, 454 patients were included and compared with 315 patients from the pre-defined SYNTAX PCI group and 334 patients from the pre-defined SYNTAX CABG cohort. At 5 years, MACCE (composite of all-cause death, stroke, any MI and any revascularization) occurred in 21.5% of SYNTAX II patients—significantly lower than in the SYNTAX PCI group—36.4% (HR 0.54; 95% CI 0.41–0.71; P < 0.001). All MACCE components, except stroke, were significantly lower in SYNTAX II PCI patients. Outcomes from SYNTAXES trial showed that HT decisions requires more than an angiographically-based algorithms. What is more, incidence of overall mortality was suboptimal in SYNTAXES even in the CABG patients, and modified OMT to improve prognosis is highly desirable, especially in diabetic patients. Continuing this plot, it should be noted that in our study more than two-fold higher rates of RR in PCI group should be weighed against the undeniably more advanced invasiveness of CABG involving greater tissue traumatization, increased risk of infection, and longer stay in intensive care unit (ICU). A greater invasiveness of CABG also generates a higher rate of cerebrovascular events in patients treated with surgery. In our work we demonstrated the confirmation of these relationships—PCI approach was found to be superior to CABG regarding the duration of postprocedural hospital stay and the incidence of strokes—both 2-times lower than in CABG-cohort, P < 0.01. In the study of Head SJ., et al.—individual patient-data pooled analysis of 11 RCTs—the largest meta-analysis so far—comparing stroke rates following surgical versus PCI revascularization, the rates of stroke at 30 days was significantly higher after PCI than CABG [0.4% vs. 1.1% (HR 0.33; 95% CI 0.20 to 0.53; P < 0.001)]. At 5-years follow-up, stroke rates remained significantly lower after PCI than after CABG (2.6% vs. 3.2%; HR 0.77; 95% CI 0.61–0.97; P = 0.027). No significant interactions between treatment and baseline clinical or angiographic variables for the 5-years rate of stroke were present, except for diabetic (PCI: 2.6% vs. CABG: 4.9%) and nondiabetic patients (PCI: 2.6% vs. CABG: 2.4%) (P for interaction = 0.004). Furthermore, individuals who experienced a stroke within 30 days of the CABG or PCI had significantly higher 5-year mortality versus those without a stroke, both after PCI (45.7% vs. 11.1%, P < 0.001) and CABG (41.5% vs. 8.9%, P < 0.001)^18,29^. What is important, the results from our real-life study, are consistent with outcomes from mentioned above meta-analysis and RTCs.

Another issue that should be addressed is the influence of the HT on decision-making process regarding revascularization in patients with MVD. Although a multidisciplinary approach in such individuals seems intuitive, the evidence in the literature confirming the validity of such proceeding are still scarce. The commonly used preprocedural risk scales—EuroSCORE II or STS do not seem to keep up with the aging population, the severity of MVD or new innovative percutaneous and surgical methods. Many factors not included in the classical risk scales as frailty, poor mobility or patient preferences have an undeniable impact on treatment results, which adds to our results a real clinical value. In our experience, one of the main challenging problem for HT is management of critically ill or hemodynamically unstable patients who need immediate decision and urgent revascularization. In our registry, 3.4% of participants had presented with cardiogenic shock. What is worth noting, more than a third (42.2%) were presented to HT due to ACS—despite the fact they need urgent management—were carefully discussed by HT.

Considering decisions made by our HT, for younger patients, but with significant LM disease, we tended to choose CABG, while for those with severe symptoms of HF, LV dysfunction or with more comorbidities PCI was preferred. These approach reflect recommendations and general perception of medical community, which support better long-term outcomes associated with CABG and greater safety of PCI for highly burdened patients. Interestingly, we did not observe a trend in choosing surgery for diabetic patients, and even statistically insignificant, but a greater percentage of these individuals received PCI. This is somewhat different from the current guidelines for myocardial revascularization^22^, but this discrepancy may be explained by the fact that cohort of patients consulted by our internal HT is not representative for the overall MVD-population. Furthermore, we attach great importance to the preferences of patients who were often afraid of open surgery. There are several methodological strengths in our study that highlight the validity of the obtained results: all-comer nature, retrospective enrolment, systematic and meticulous patient assessment, complete mean 60-months clinical follow-up, assessment of quality of life and the use of standardized definitions and clearly defined endpoints. Undoubtedly, the greatest advantage of this study is an enrollment of real-life patients, while the previous trials have noted the disqualification of a significant percentage of population with MVD due to lack of consent or failure to meet inclusion/exclusion criteria. Furthermore, a large group of patients included into this single-centre registry and complete long-term follow-up are sufficient to demonstrate that implemented decisions are consistent with clinical practice. Properly selected endpoints, reflecting the most common and serious complications of interventional strategies, prove the translatability of the obtained results on proper functioning of HT.

## Conclusions

In this single-centre, retrospective study we presented how the HT approach and implemented decisions influenced the outcomes and quality of life of MVD-patients, demonstrating that there were no statistically significant differences in long-term survival between patients qualified for surgery or percutaneous approach. To summarize, it should be emphasized that for patients with severe CAD, the choice of the optimal treatment strategy should never be an individual, and only HT seems to be an appropriate tool to ensure satisfactory long-term outcomes and an acceptable quality of life. Further research, including RCTs are required to establish the cooperation of HT, but our preliminary results may provide a cornerstone for the future, underlining that the role of HT should be emphasized both in clinical practice and in guidelines for patients with MVD.

### Limitations

Nevertheless, this study should be considered with the following limitations. The first and the most important is its retrospective, non-randomized character and single-centre design. Above that, the decisions-making process have to be assigned to our internal, individual HT cooperation and cannot be interpreted as a general one. Furthermore, regular use of medications by patients undergoing coronary revascularization has a significant impact on prognosis, but is not measurable and usually remains a matter of trust, therefore the reliability of the achieved endpoints may be questioned. Moreover, patients with non-implemented decisions were not included into final analysis, so the achieved follow-up may slightly differ from reality. Patients were not matched, hence comparison of groups should be considered with caution. Summarizing, due to the nature of observational study, patients qualified for CABG or PCI differed significantly in some clinical parameters, therefore the obtained outcomes cannot be used as a basis for formulating far-reaching and indisputable conclusions.

## Data Availability

The data presented in this study are available on request from the corresponding author. The data are not publicly available due to any accessible repository.
